# CSF1R inhibition for histiocytic neoplasm with *CBL* mutations refractory to MEK1/2 inhibition

**DOI:** 10.1038/s41375-023-01947-4

**Published:** 2023-06-24

**Authors:** Eli L. Diamond, Jasmine H. Francis, Mario E. Lacouture, Veronica Rotemberg, Mariko Yabe, Kseniya Petrova-Drus, Gary A. Ulaner, Ryan Reddy, Omar Abdel-Wahab, Benjamin H. Durham

**Affiliations:** 1 Department of Neurology, Memorial Sloan Kettering Cancer Center, New York, NY USA; 2 Ophthalmic Oncology Service, Department of Surgery, Memorial Sloan Kettering Cancer Center, New York, NY USA; 3 Dermatology Service, Department of Medicine, Memorial Sloan Kettering Cancer Center, New York, NY USA; 4 Department of Pathology and Laboratory Medicine, Memorial Sloan Kettering Cancer Center, New York, NY USA; 5 Department of Molecular Imaging and Therapy, Hoag Family Cancer Institute, Irvine, CA USA; 6 Molecular Imaging and Therapy Service, Department of Radiology, Memorial Sloan Kettering Cancer Center, New York, NY USA; 7 Molecular Pharmacology Program, Sloan Kettering Institute, Memorial Sloan Kettering Cancer Center, New York, NY USA

**Keywords:** Oncogenes, Targeted therapies

## To the Editor:

Histiocytic neoplasms are rare clonal hematopoietic disorders occupying a wide clinical spectrum amongst adults and children. Identification of recurrent somatic mutations in the mitogen activated protein kinase (MAPK) pathway has reframed our understanding of these diseases from autoimmune conditions to clonal myeloid neoplasms. Equally important knowledge of mutational drivers of histiocytoses has overhauled the clinical management of these disorders. Erdheim-Chester disease (ECD) is a multisystem histiocytic neoplasm affecting adults characterized by osseous infiltration in the distal legs and variable involvement of retroperitoneal, cardiovascular, cutaneous, neurological, and other structures [1]. More than 50% of ECD lesions harbor *BRAF*^V600E^ mutations, and in 2018 vemurafenib was approved by the U.S. Food and Drug Administration (FDA) for *BRAF*^V600E^-mutated ECD [2]. The MEK1/2 inhibitor, cobimetinib, was recently broadly approved for the treatment of histiocytic neoplasms based on a phase 2 trial in which 20/24 patients with diverse histiocytic neoplasms including mutations in BRAF, N/KRAS, and MEK1/2 demonstrated a complete or partial response by [^18^F]-Fluorodeoxyglucose positron-emission tomography (FDG-PET).

Recent studies have identified a subset of histiocytic neoplasm patients with activating mutations upstream of RAS-RAF-MEK1/2 including in the receptor tyrosine kinase CSF1R [3]. Importantly, one patient with an activating *CSF1R* mutation has been treated successfully with the CSF1R inhibitor, pexidartinib [4]. Given the requirement of CSF1R signaling for development and maintenance of the monocyte/macrophage lineage and the observation that CSF1R is required for differentiation of lesional cells within LCH [5], it has been hypothesized that CSF1R inhibition may be efficacious in broader genetic subtypes of histiocytosis patients. To date, however, evaluation of CSF1R inhibition in histiocytic neoplasms outside of one patient with CSF1R-mutated disease has not been explored. We present here a case of ECD with progression despite MEK inhibition with a dramatic and lasting response to CSF1R inhibition.

**Fig. 1:**
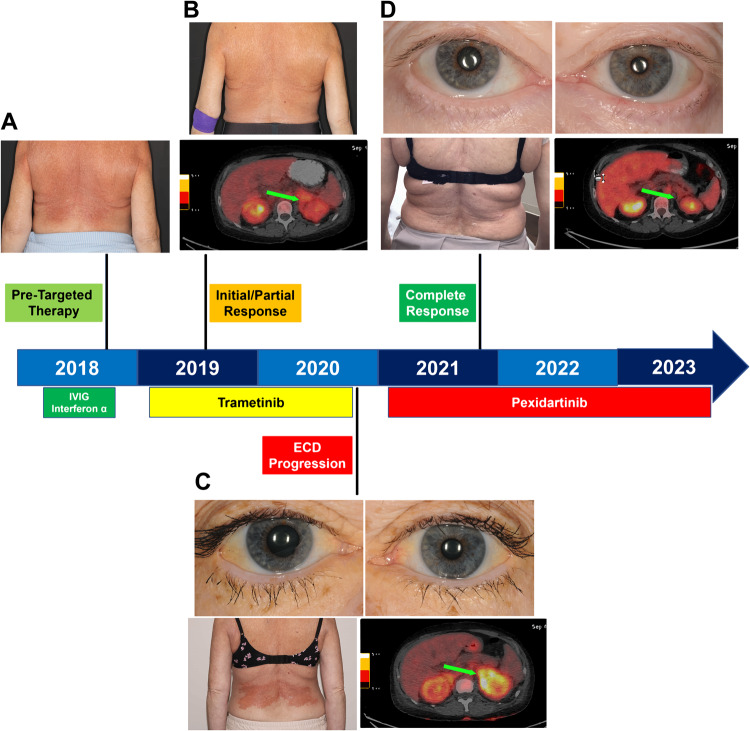
Clinical Responses to MEK inhibition and CSF1R inhibition in CBL-mutated histiocytic neoplasm. Biopsy-proven histiocytic neoplasm manifest clinically as diffuse erythematous rash and renal mass (green arrows). Disease is demonstrated clinically prior to targeted therapy (**A**), with initial clinical and radiological responses to MEK inhibition in the skin and retroperitoneum (**B**), progression of disease on MEK inhibition with recurrence of rash, peri-renal lesion, and emergence of episcleral and peri-ocular disease (**C**), and complete response to pexidartinib in all disease sites (**D**) with a schematic of the patient’s therapy and response timeline.

**Fig. 2:**
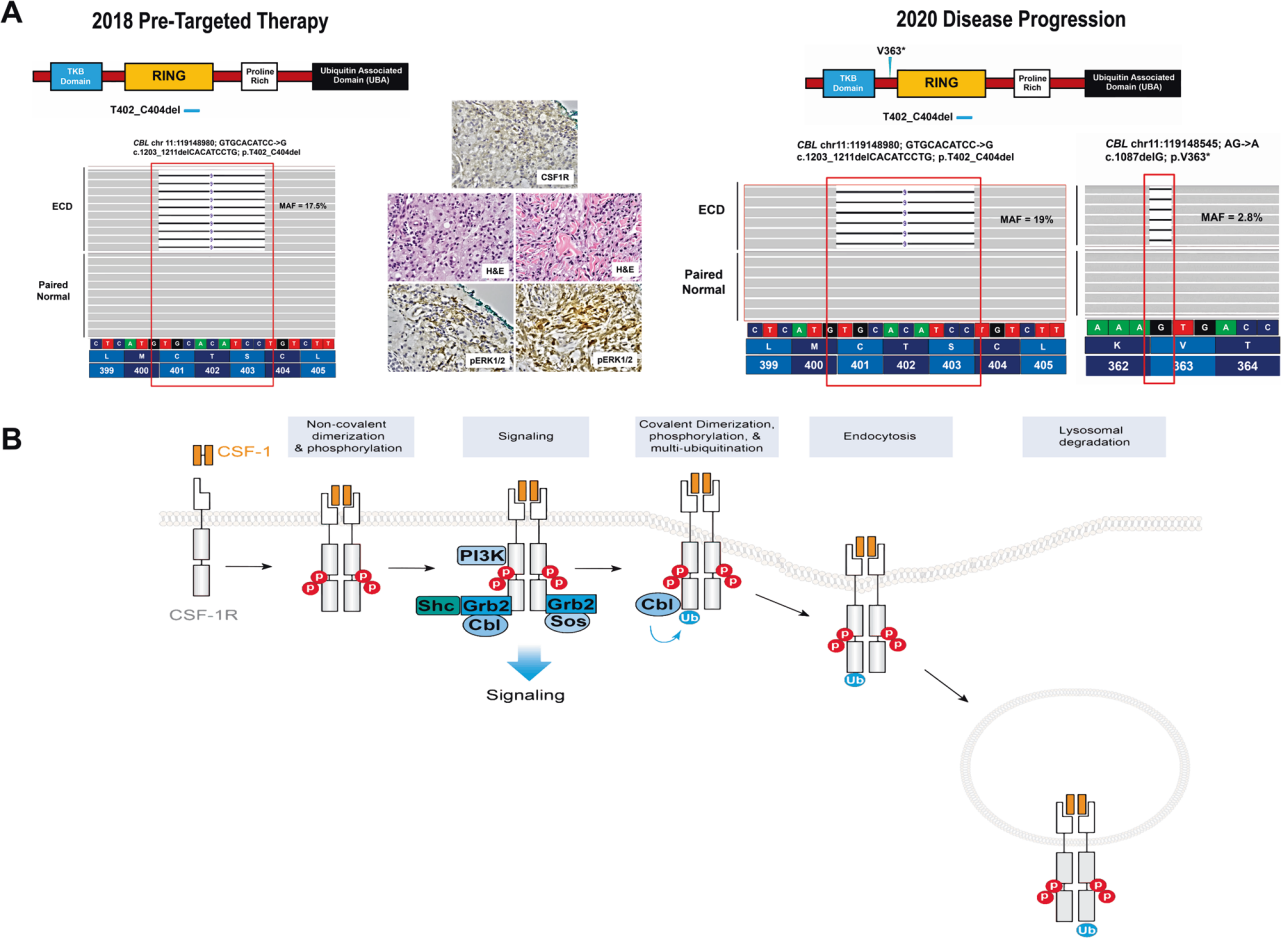
Pathological analysis of *CBL*-mutated histiocytosis patient and an overview of CBL-regulation of CSF1-R surface protein expression. **A** A somatic *CBL* exon 8; c.1203_1211delCACATCCTG; p.T402_C404del 9 bp in-frame deletion involving the RING finger domain of CBL was detected by clinical targeted next-generation sequencing (NGS) of the Erdheim-Chester Disease (ECD) involving the patient’s 2018 skin biopsy. The neoplastic histiocytes in the 2018 skin biopsy (left side) showed weak surface immunoreactivity for CSF1-R (CD115) and overexpression of phospho-ERK1/2 prior to trametinib therapy. A new skin lesional biopsy in 2020 while the patient was on trametinib (right side) also showed non-Langerhans cell histiocytosis with weak surface immunoreactivity for CSF1-R (CD115) and strong overexpression of phospho-ERK1/2 consistent with a recurrence of ECD (CSF1-R Immunohistochemistry; Hematoxylin and Eosin; phospho-ERK1/2 immunohistochemistry; 600x magnification). Targeted NGS of the 2020 ECD skin lesion demonstrated the previously identified *CBL* p.T402_C404del in-frame deletion as well as a new, sub-clonal somatic *CBL* exon 7; c.1087delG; p.V363* nonsense (truncating) mutation involving the linker region of the CBL protein prior to the RING finger domain. **B** CSF-1 binding to CSF-1R results in non-covalent dimerization and phosphorylation of CSF-1R and subsequent phosphorylation of CBL, SHC, the p85 subunit of PI3K and their association with CSF-1R and GRB2. Membrane bound CBL then results in ubiquitinylation of CSF-1R which is associated with internalization and degradation of the ubiquitinylated form of CSF-1R.

A 58-year-old woman with a past medical history of hypertension and chronic gastritis presented with 3 years of progressive fatigue, diffuse edema, and bone pain in the legs. Physical examination demonstrated pitting edema in the legs, diffuse rash consisting of partially blanching large red patches on the chest and back, and periorbital xanthelasmas (Fig. 1). Laboratory studies demonstrated normocytic anemia (Hb 9.4 g/dL), lymphopenia (absolute lymphocyte count 0.8 K/µL), and otherwise normal blood counts, hypoalbuminemia (2.1 g/dL), and elevated C-reactive protein (7.0 mg/dL). Computed tomography (CT) and full-body FDG-PET/CT demonstrated widespread FDG-avid infiltrates in the bones of the arms and legs, the retroperitoneum, mediastinum, para-aortic regions, pericardium, and pleura. Biopsy of retroperitoneal infiltrates, chest rash, and xanthelasma demonstrated a xanthomatous histiocytic lesion immunoreactive for CD68, CSF1-R (CD115), overexpressing phospho-ERK1/2, and without positivity for CD1a, S100, or CD207 (Langerin), confirming a non-Langerhans cell neoplasm (non-LCH) (Fig. 2A). The constellation of non-LCH with involvement of the distal extremities, retroperitoneum, and para-aortic structures established a diagnosis of ECD. A large next-generation sequencing (NGS) panel of all protein-coding exons and selected introns of 576 genes known to undergo somatic genomic alterations in hematological neoplasms was performed on three separate biopsies from 2018 and did not demonstrate mutations in *BRAF* or other MAPK pathway genes. Rather, these biopsies demonstrated an in-frame deletion in exon 8 encoding the RING finger domain of CBL (*CBL* c.1203_1211delCACATCCTG; p.T402_C404del) (Fig. 2A).

The patient was treated briefly with intravenous immune globulin, as well as interferon-alpha, both without clinical benefit. She was treated next with the MEK inhibitor trametinib at 1 mg orally daily with regression of infiltrates by FDG-PET/CT and resolution of rash (Fig. 1). However, after 18 months of treatment, the erythematous rash re-emerged in the same distribution, and FDG-PET/CT demonstrated metabolic recurrence of multifocal infiltrative lesions. Physical examination demonstrated yellow plaques on the episclera, indicating progression of ocular disease (Fig. 1). Skin biopsy in 2020 again demonstrated non-LCH with greater overexpression of phospho-ERK1/2 than in the 2018 lesional biopsy, and repeated NGS demonstrated the previously identified *CBL* in-frame deletion as well as a new, sub-clonal nonsense mutation in exon 7 involving the linker domain of CBL (*CBL* c.1087delG; p.V363*) (Fig. 2A).

Treatment was initiated with pexidartinib 200 mg orally twice daily through commercial drug access. Dramatic regression in rash, resolution of episcleral disease and xanthelasmas, as well as resolution of FDG-avid disease by PET/CT, was observed after 4 months of treatment (Fig. 1). The patient has completed 24 months of treatment with a sustained complete response. No hepatotoxicity or other adverse events have been observed. As the optimal duration of treatment in ECD with targeted therapies remains unknown, the patient currently faces indefinite treatment.

The U.S. FDA approval of cobimetinib for adults with histiocytic neoplasms has provided an important advancement for patients with these disorders. At the same time, histiocytosis patients with acquired resistance to MEK inhibitors or intolerant to these agents are beginning to be observed. Here we identify acquired mutations in the E3 ubiquitin ligase CBL as associated with resistance to MEK inhibition and progression of disease in a histiocytosis patient. Given the knowledge that CBL stimulates CSF1R multi-ubiquitination and endocytosis to limit macrophage proliferation [6] (Fig. 2B), we hypothesize that the loss of the normal CBL proteins within the neoplastic histiocytes resulted in the absence of cellular recycling of CSF1R through its physiological ubiquitination and that this resulted in persistent or sustained activation of the receptor to a degree that was no longer inhibited by the MAPK pathway inhibition achieved with trametinib. This finding of resistance to MEK inhibition with CBL loss is consistent with CBL’s role as a haploinsufficient tumor suppressor in myeloid neoplasms in which transformation to leukemia is most frequently seen upon loss-of-heterozygosity of a single initiating *CBL* mutation [7, 8]. Lack of response to trametinib following acquisition of both the somatic *CBL* mutations in a subset of neoplastic histiocytes was demonstrated with greater overexpression of phospho-ERK1/2 in the 2020 biopsy at disease progression than the initial 2018 biopsy prior to the initiation of targeted therapy. Potentially consistent with this hypothesis, CSF1R inhibition resulted in clinical benefit in this patient following failure of trametinib therapy. Overall, these data suggest a broader potential role for CSF1R inhibition in histiocytosis patients beyond patients with CSF1R activating mutations.
